# Silicosis as a predictor of tuberculosis mortality and treatment failure and need for incorporation in differentiated TB care models in India

**DOI:** 10.1186/s13690-023-01189-x

**Published:** 2023-09-26

**Authors:** Mihir P. Rupani

**Affiliations:** Clinical Epidemiology (Division of Health Sciences), ICMR - National Institute of Occupational Health (NIOH), Indian Council of Medical Research, Meghaninagar , Ahmedabad City, Gujarat 380016 India

**Keywords:** Determinants, Correlates, Death, Default, Silicosis, Silico-tuberculosis, Silica dust, India

## Abstract

**Background:**

Differentiated tuberculosis (TB) care is an approach to improve treatment outcomes by tailoring TB management to the particular needs of patient groups based on their risk profile and comorbidities. In silicosis-prone areas, the coexistence of TB and silicosis may exacerbate treatment outcomes. The objective of the study was to determine predictors of TB-related mortality, treatment failure, and loss to follow-up in a silicosis-prone region of western India.

**Methods:**

A retrospective cohort was conducted among 2748 people with TB registered between January 2006 and February 2022 in Khambhat, a silicosis-prone block in western India. Death, treatment failure, and loss to follow up were the outcome variables. The significant predictors of each outcome variable were determined using multivariable logistic regression and reported as adjusted odds ratios (aOR) with 95% confidence intervals (CIs).

**Results:**

In the cohort of 2,748 people with TB, 5% presented with silicosis, 11% succumbed to the disease, 5% were lost to follow-up during treatment, and 2% encountered treatment failure upon completion of therapy. On multivariable logistic regression, concomitant silicosis [aOR 2.3 (95% CI 1.5–3.5)], advancing age [aOR 1.03 (95% CI 1.02–1.04)], male gender [aOR 1.4 (95% 1.1–1.9)], human immunodeficiency virus (HIV) positive [aOR 2.2 (95% 1.02–4.6)], and previous TB treatment [aOR 1.5 (95% CI 1.1–1.9)] significantly predicted mortality among people with TB. Concomitant silicosis [aOR 3 (95% CI 1.4–6.5)], previous TB treatment [aOR 3 (95% CI 2–6)], and multi-drug resistant TB [aOR 18 (95% CI 8–41)] were the significant predictors of treatment failure on adjusted analysis. Advancing age [aOR 1.012 (1.001–1.023)], diabetes [aOR 0.6 (0.4–0.8)], and multi-drug resistance [aOR 6 (95% CI 3–12)] significantly predicted loss to follow-up after adjusting for confounders.

**Conclusions:**

Controlling silicosis might decrease TB mortality and treatment failure in silicosis-prone regions. The coexistence of HIV and silicosis may point to an increase in TB deaths in silicosis-prone areas. Silicosis should now be acknowledged as a major comorbidity of TB and should be included as one of the key risk factors in the differentiated TB care approach. Primary care physicians should have a high clinical suspicion for silicosis among individuals diagnosed with TB in silicosis-prone blocks.

**Table:** 

Text box 1. Contributions to the literature
• Reveals, for the first time, a significant association between silicosis and tuberculosis (TB) treatment failure, shedding light on a critical issue in TB management.
• Urges extra vigilance among doctors in silica-exposed TB regions to spot potential silicosis cases.
• Emphasizes that when TB and silicosis coexist, it increases the risk of death and makes TB treatment less effective.
• Advocates for special care for those with both TB and silicosis, including dust avoidance and early TB drug resistance testing.
• Provides vital insights into the intricate factors affecting TB outcomes in silicosis-prone areas, advancing our understanding in addressing the dual burden of TB and silicosis.

## Introduction

In 2021, there were 6.4 million reported cases and 1.6 million deaths due to tuberculosis (TB) worldwide [1]. India had the highest number of reported TB cases at 2.42 million and the highest number of reported TB deaths at 0.5 million in 2022 [1, 2]. Countries have resolved to eliminate TB by the year 2030, with India resolving to achieve this feat by the year 2025 [3, 4]. Reduction in incidence, mortality, and catastrophic costs due to TB are the elimination targets set under the End-TB strategy [3]. Death while on TB treatment, testing positive at the end of TB treatment, and interrupting TB treatment for ≥ 1 month are considered unfavorable treatment outcomes of TB under the national TB program of India [5]. In resource-limited settings, a differentiated TB care approach identifies patients with a high risk of severe illness due to factors such as comorbidities and triage them for comprehensive assessment, and inpatient care [6]. This strategy aims to reduce the incidence of TB-related deaths in such settings [7].

Among several vulnerable populations of TB, patients with silicosis are now recognized as a vulnerable population for presumptive TB [8]. Silicosis is one of the commonest occupational disease worldwide since over decades, accounting for 39% of all pneumoconiosis globally in 2017 [9, 10]. In 2019, 2.65 million global prevalent cases of silicosis have been reported with an incidence rate and death rate of 1.7 and 0.2 per 100,000 population respectively [11]. The prevalent cases and deaths due to silicosis might be much higher as issues related to probable underreporting of occupational diseases have been sufficiently highlighted [12–15].

Human immunodeficiency virus (HIV) infection, increasing age, tobacco smoking, alcohol drinking, low income, stigma, and retreatment are some of the predictors of death, treatment failure, and loss to follow-up described in various studies [16–18]. Silicosis, along with increasing the risk for developing TB, is also associated with death and treatment failure among people with TB [19]. Several studies have researched the predictors of failing to complete TB treatment, however, an adjusted analysis of various predictors in the presence of silicosis is lacking.

Certain occupations, such as mining, construction, cement, stone cutting, foundries, and quarries, are mainly implicated in causing silicosis [9]. As exposure to silica dust is the primary reason for the causation of silicosis, the burden of silicosis is localized to certain blocks where industries generating silica dust are located. It is hypothesized that silicosis, in conjunction with other predictors such as HIV, retreatment, and multi-drug resistance, may worsen TB treatment outcomes. There is a paucity of evidence on the predictors of unfavorable treatment outcomes of TB in silicosis-prone blocks. In the context of this study, we underscore the significance of adopting a 'differentiated care' approach to TB management. We advocate for intensified care strategies tailored to individuals with silicosis from the outset of TB treatment, recognizing their heightened vulnerability to unfavorable treatment outcomes. The objective of the study was to determine the predictors of mortality, treatment failure, and loss to follow-up due to TB in a silicosis-prone block in western India.

## Methods

### Study design

**Fig. 1:**
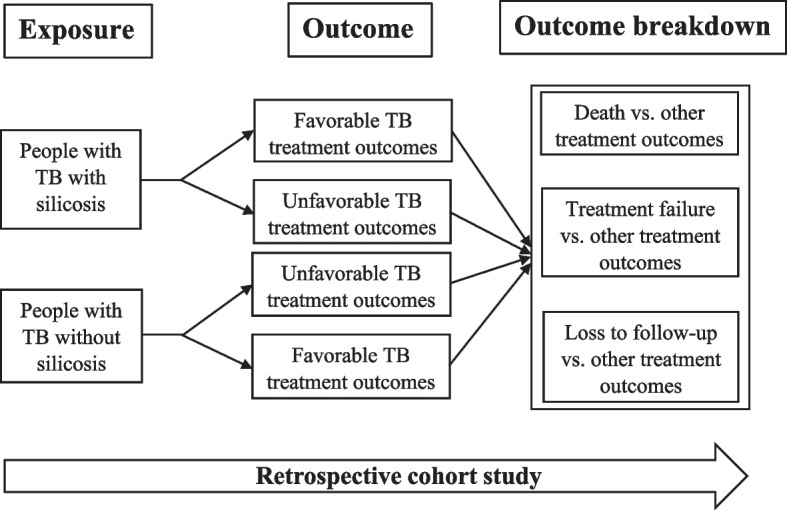
Schema of retrospective cohort study for predictors of mortality, treatment failure, and loss to follow-up due to TB in Khambhat block

This study represents a retrospective cohort design, which builds upon the investigator’s prior work [19], the key distinction lies in the approach to analyzing unfavorable outcomes (Fig. 1). Instead of examining these outcomes as a composite, as done in the prior publication, this analysis investigates individual types of unfavorable outcomes (death, treatment failure, and loss to follow-up). This distinction underscores the focus on assessing distinct treatment outcomes within the context of a retrospective cohort study [19]. The primary article includes all details of the methods and selection of the people with TB [19]. The retrospective cohort determined the association of silicosis with unfavorable treatment outcomes of TB in Khambhat block, a silicosis-prone block in Gujarat state in western India [19].

### Study setting, duration, and population

Khambhat block is known for crushing, cutting, and polishing of agate stones, which contain a significant quantity (60%) of crystalline silica, and is one of the places in Gujarat reporting silicosis [20–24]. For the present study, a retrospective cohort study was designed using individual unfavorable treatment outcomes as the outcome variables, with treatment outcome assignment by the national TB program as the end point. In the current study, we included people with TB from the Khambhat block. The data was extracted from the Nikshay online portal and physical treatment registers of people with TB in Khambhat block between January 2006 and February 2022 [19]. To clarify, all predictors for this study were indeed measured at baseline, specifically at the initiation of TB treatment. It is important to note that the data were extracted from Nikshay/ physical registers rather than being primarily collected.

### Variables

#### Outcome variables

The dichotomous outcome variables for this study were death due to TB, treatment failure, and loss to follow-up, all of which are considered unsuccessful treatment outcomes of TB under the national TB program [5]. TB-related mortality was characterized by the occurrence of patient death during the course of anti-TB treatment [5]. Treatment failure was defined as the persistence of a positive biological specimen by smear or culture at the conclusion of the treatment regimen [5]. ‘Loss to follow-up’ was attributed to people with TB whose treatment was discontinued for a duration exceeding one month [5].

#### Exposure variable

The exposure variable was people with TB with silicosis vs. those without silicosis.

#### Predictor variables

Age (years), gender, weight (kilograms), human immunodeficiency virus (HIV) infection status, diabetes status, sputum positivity for TB, previously treated for TB (current treatment after treatment failure or treatment interruption in previous episode of TB), extra-pulmonary TB, multi-drug resistance, and relapsed for TB (current treatment after declared cured or treatment completed in previous episode of TB) were the predictor variables.

### Statistical analysis

Continuous variables were depicted by the median (interquartile range IQR), whereas categorical variables were expressed as percentages. Uni-variable logistic regression was applied for each of the three outcome variables (death due to TB, treatment failure, and lost to follow-up) in conjunction with both the exposure variable and individual predictor variables. The multivariable logistic regression model included variables with *p*-values ≤ 0.2. To identify significant predictors of each of the three outcome variables, multivariable logistic regression was used. As ‘previously treated for TB’ and ‘relapsed for TB’ demonstrated multi-co-linearity, the former variable was retained in the final model [19]. On multivariable analysis, predictors were considered significant when the *p*-value was < 0.05. The adjusted odds ratios (with 95% confidence intervals CIs) of significant predictors were presented.

## Results

### Characteristics of people with TB

The median (IQR) age of the 2748 people with TB was 42 (30–55) years, 66% were male, 2% each had HIV infection and multi-drug resistance, 23% were previously treated for TB, and 5% had concomitant silicosis (Table 1). In addition, 18% of the people with TB had adverse treatment outcomes: 11% died, 5% were lost to follow-up during treatment, and 2% suffered treatment failure at the end of treatment.

**Table 1:** Characteristics of people with TB with assigned TB treatment outcomes in Khambhat block (*n* = 2748)

Characteristics	Number (%) or median (IQR)
Age (years)	42 (30–55)
Male gender	1825 (66)
Weight (kilograms) (*n* = 829)	42 (36–49)
HIV positive	45 (2)
Diabetic	905 (33)
Sputum positive TB	1513 (55)
Previously treated for TB	642 (23)
Extra-pulmonary TB	327 (12)
Multi-drug resistant TB	61 (2)
Relapsed for TB	589 (21)
Concomitant silicosis	138 (5)
Treatment outcomes of TB	
Successful	
Cured	992 (36)
Treatment completed	1247 (46)
Unfavorable	
Treatment failure	54 (2)
Lost to follow-up	144 (5)
Died	311 (11)

*HIV* Human immunodeficiency virus, *TB* Tuberculosis

### Predictors of mortality

On multivariable logistic regression analysis, concomitant silicosis, age (in years), male gender, HIV positive, and previous TB treatment significantly predicted mortality among people with TB (Table 2). Concomitant silicosis was associated with 2.3 (95% CI 1.5–3.5) times higher odds, males with 1.4 (95% 1.1–1.9) times higher odds, those with HIV with 2.2 (95% 1.02–4.6) times higher odds, and previous TB treatment was associated with 1.5 (95% CI 1.1–1.9) times higher odds of mortality due to TB. A one-year increase in age was associated with a 3% (95% CI 2%-4%) higher odds of mortality due to TB.

**Table 2:** Uni-variable and multivariable^a^ logistic regression for predictors of mortality due to TB in Khambhat block (*n* = 2748)

Variables	Crude OR (95% CI)	*p*-value	Adjusted OR (95% CI)	*p*-value
Concomitant silicosis	2.5 (1.7–3.8)	< 0.001	2.3 (1.5–3.5)	** < 0.001**
Age (years)	1.03 (1.02–1.04)	< 0.001	1.03 (1.02–1.04)	** < 0.001**
Male gender	1.6 (1.3–2.2)	< 0.001	1.4 (1.1–1.9)	**0.016**
Weight (*n* = 829)	0.98 (0.96–1.003)	0.081	-	-
HIV positive	1.9 (0.9–4.2)	0.069	2.2 (1.02–4.6)	**0.044**
Diabetes	0.9 (0.7–1.2)	0.487	-	-
Sputum positive TB	1.2 (0.9–1.6)	0.096	1.2 (0.9–1.6)	0.189
Previously treated for TB	1.6 (1.3–2.1)	< 0.001	1.5 (1.1–1.9)	**0.006**
Extra-pulmonary TB	0.7 (0.4–1.02)	0.064	0.9 (0.6–1.3)	0.504
Multi-drug resistant TB	0.9 (0.4–1.9)	0.712	-	-

*HIV* Human immunodeficiency virus, *TB* Tuberculosis, *OR* Odds ratio, *CI* Confidence intervals

^a^Model statistics: Omnibus chi-square = 93 (*p*-value < 0.001); Hosmer–Lemeshow *p*-value = 0.489; Nagelkerke r^2^ = 0.065; classification accuracy = 89%

### Predictors of treatment failure

Concomitant silicosis, previous TB treatment, and multi-drug resistant TB were the significant predictors of treatment failure on multivariable logistic regression (Table 3). Those with concomitant silicosis had 3 (95% CI 1.4–6.5) times higher odds of TB treatment failure, those previously treated for TB had 3 (95% CI 2–6) times higher odds, and those having multi-drug resistant TB had 18 (95% CI 8–41) times higher odds.

**Table 3:** Uni-variable^a^ and multivariable^b^ logistic regression for predictors of treatment failure of TB in Khambhat block (*n* = 2748)

Variables	Crude OR (95% CI)	*p*-value	Adjusted OR (95% CI)	*p*-value
Concomitant silicosis	5 (3–10)	< 0.001	3 (1.4–6.5)	**0.005**
Age (years)	0.9 (0.9–1)	0.448	-	**-**
Male gender	2.3 (1.1–4.5)	0.021	2 (0.9–3.8)	0.076
Weight (*n* = 829)	1.01 (0.9–1.1)	0.692	-	-
HIV positive	1.1 (0.2–8.4)	0.900	-	-
Diabetes	1.5 (0.9–2.6)	0.130	1.4 (0.8–2.6)	0.218
Previously treated for TB	2.5 (1.4–4.3)	0.001	3 (2–6)	** < 0.001**
Multi-drug resistant TB	15 (8–31)	< 0.001	18 (8–41)	** < 0.001**

*HIV* Human immunodeficiency virus, *TB* Tuberculosis, *OR* Odds ratio, *CI* Confidence intervals

^a^sputum positive TB and extra-pulmonary TB were removed from the analysis due to lack of data in the comparison group

^b^Model statistics: Omnibus chi-square = 68 (*p*-value < 0.001); Hosmer–Lemeshow *p*-value = 0.279; Nagelkerke r^2^ = 0.139; classification accuracy = 98%

### Predictors of loss to follow-up

On multivariable analysis, age (in years), diabetes, and multi-drug resistance significantly predicted loss to follow-up among people with TB (Table 4). A one-year increase in age raised the chances of loss to follow-up by 1.2% (95% CI 0.1%-2.3%). When compared to drug-sensitive TB, multi-drug resistant TB increased the odds of loss to follow-up by 6 (95% CI 3–12) times. Being a diabetic reduced the chances of TB-related loss to follow-up by 40% (95% CI 20%-60%).

**Table 4:** Uni-variable and multivariable^a^ logistic regression for predictors of loss to follow-up due to TB in Khambhat block (*n* = 2748)

Variables	Crude OR (95% CI)	*p*-value	Adjusted OR (95% CI)	*p*-value
Concomitant silicosis	1.6 (0.9–3.1)	0.143	1.1 (0.6–2.2)	0.696
Age (years)	1.01 (0.99–1.02)	0.066	1.012 (1.001–1.023)	**0.036**
Male gender	1.4 (0.9–2.0)	0.091	1.3 (0.9–1.8)	0.248
Weight (*n* = 829)	0.99 (0.95–1.03)	0.634	-	-
HIV positive	1.3 (0.4–4.2)	0.666	-	-
Diabetes	0.6 (0.4–0.9)	0.015	0.6 (0.4–0.8)	**0.005**
Sputum positive TB	1.5 (1.04–2.09)	0.030	1.2 (0.8–1.8)	0.317
Previously treated for TB	1.2 (0.8–1.7)	0.378	-	-
Extra-pulmonary TB	0.4 (0.2–0.8)	0.010	0.5 (0.2–1.1)	0.079
Multi-drug resistant TB	6 (3–11)	< 0.001	6 (3–12)	** < 0.001**

*HIV* Human immunodeficiency virus, *TB* Tuberculosis, *OR* Odds ratio, *CI* Confidence intervals

^a^Model statistics: Omnibus chi-square = 45 (*p*-value < 0.001); Hosmer–Lemeshow *p*-value = 0.571; Nagelkerke r^2^ = 0.049; classification accuracy = 95%

## Discussion

To summarize, concomitant silicosis and previous TB treatment predicted mortality and treatment failure, but not ‘lost to follow-up’ among people with TB in a silicosis-prone block. Moreover, being male and HIV-positive predicted mortality due to TB, whereas multi-drug resistance predicted both treatment failure and loss to follow up due to TB. In my study setting, advancing age increased the chances of death and loss to follow-up.

Among the three unfavorable TB treatment outcomes, mortality and treatment failure can be considered as ‘biological’ outcomes, reflecting the physiological impact of the diseases, while loss to follow-up is better characterized as a ‘behavioral’ or 'access-related' outcome, influenced by patient actions and healthcare access. It is noteworthy that silicosis and prior TB infection did not significantly impact the likelihood of loss to follow-up, as this outcome is influenced more by patient behavior and access to healthcare. In fact, having experienced a previous episode of TB may heighten peoples’ awareness of the importance of completing treatment. On the other hand, older males with risk factors such as silicosis and HIV exhibited a higher likelihood of mortality compared to their counterparts, underscoring the complex and synergistic interactions that multiple comorbidities can have among people with TB, impacting their overall health. The extended treatment duration for multi-drug resistant TB may have contributed to an increased risk of loss to follow-up, while the intricate treatment regimen involving multiple drugs may have played a more direct role in raising the chances of treatment failure. Additionally, silicosis-related lung fibrosis could potentially contribute to both treatment failure and mortality among people with TB. This diversity in predictors underscores the importance of adopting differentiated care strategies that precisely address the unique risk factors associated with each unfavorable treatment outcome. By doing so, we can aim to enhance overall treatment outcomes and mitigate the dual burden that TB and silicosis pose on the lungs.

Several occupational, environmental, and biological factors might explain my study’s findings. People with TB in my research lived in an area where they had been exposed to silica dust as a result of agate stone-related occupational activities [20–24]. In Khambhat block, larger stones are chipped, baked, crushed/ground, and then polished/drilled at the household-level, with the grinding process producing the most dust [22, 24]. Agate stone-related work is the principal occupation of the people residing in certain villages of the Khambhat block [22]. Due to the cottage nature of the agate stone industry, family members of employees directly involved in agate stone-related activity may also be exposed to silica dust non-occupationally [22, 24, 25]. Silica dust exposure generates reactive oxygen species and causes alveolar cell death [26], resulting in immunological dysfunction and an increased likelihood of TB bacilli persistence and relapse of TB disease [27]. When the pulmonary macrophages consume silica dust particles, they become dysfunctional, triggering the production of inflammatory markers leading to fibrosis of the lung tissue [28–30]. Silica dust-induced lung fibrosis hinders TB medications from entering into affected lung tissue [28–30], increasing the likelihood of treatment failure. I believe some of the people with TB in my research were exposed to silica dust, either occupationally or non-occupationally, but were not diagnosed with silicosis or were yet to be diagnosed with silicosis. In Khambhat block, underreporting of silicosis, a notifiable and compensable occupational disease, cannot be ruled out [12].

The study’s finding of multiple predictors such as silicosis, HIV, previous TB treatment, age, and being male predicting death among people with TB partially supports my hypothesis: concomitant silicosis, in the presence of multiple predictors, worsens TB treatment outcomes. A study among South African gold-mine workers also found that silicosis and HIV were strongly associated with an increased risk of death due to TB [31]. My study revealed more than twice the risk of TB mortality from HIV and silicosis, but South African researchers discovered 15 times and three times the risk, respectively, from HIV and silicosis [31]. Even in the absence of silicosis, HIV has been shown to be a predictor of unfavorable TB treatment outcomes [18]. All of the predictors identified to be predicting mortality among people with TB in my study (excluding silicosis) were also corroborated in a study conducted in China [32]. Age and male gender were also established as risk factors for death among people with TB in other studies in India and Ethiopia [33].

In the present study, concomitant silicosis, previous treatment for TB, and multi-drug resistance predicted the likelihood of testing positive for TB disease at the end of the treatment. HIV infection was shown to be associated with a high recurrence of TB among South African gold miners [34], however the current study found no indication of this. Experts in the area of silicosis have previously opined that silicosis or silica dust exposure are not risk factors for recurrence, relapse, or reinfection [35], but my study showed a threefold increase in the chances of treatment failure. Other researchers have found that patients with silicosis have greater likelihood of TB relapses [19, 36–38], although this finding has been disputed by another study [39]. By definition, relapse and treatment failure are not synonymous. Relapse is defined as testing positive for TB after being declared cured, whereas treatment failure is defined as testing positive for TB at the end of TB treatment [5]. Although research shows that sputum smear positive at the end of treatment is not a good indicator of TB treatment failure [40], this is the first study to document an association between silicosis and TB treatment failure.

In my study, 5% of people with TB were lost to follow up, compared to 12% of patients with silico-tuberculosis in a clinical trial in Hong Kong [36]. Adverse drug reactions have been reported to be the most prevalent cause of loss to follow up among people with TB [41]. In the clinical trial in Hong Kong, patients with silico-tuberculosis experienced 22% adverse drug reactions [36]. The current study found no association between silicosis and loss to follow-up; nevertheless, future studies should document the occurrence of adverse drug reactions in silico-tuberculosis patients and compare them to people with TB without silicosis. Also, while my study did not find an association, HIV co-infection has been identified as a predictive factor for loss to follow-up [18]. Finally, because my primary research found higher rates of multi-drug resistant TB among patients with silicosis [19], I presume that these higher rates may have contributed to the increased loss to follow-up in this study.

In the current study context of a silicosis-prone block, increasing age predicted loss to follow-up and previous TB treatment predicted treatment failure, and multi-drug resistance predicted both the unfavorable treatment outcomes. Several research have been conducted across the world to determine the predictive factors of unfavorable TB treatment outcomes [16–18, 42–48]. Similar to my study, advancing age [16, 43, 44], male gender [16, 42–44], HIV [16, 17, 43, 44], previous TB treatment [17, 44–46], and multi-drug resistance[45] were significant predictors of unfavorable TB treatment outcomes in other studies.

My research found that the presence of diabetes improved treatment adherence among people with TB, which contradicted the present understanding [47]. The only explanation for this paradoxical result is that a nationwide programme for bidirectional screening activities for TB-diabetes was initiated in India in 2017 [49]. The emphasis placed on early detection and care of both TB and diabetes might have improved the treatment adherence among people with TB in my study [49, 50]. Collaborative TB and silicosis activities have also been proposed, with an emphasis on increasing silicosis and TB surveillance in silicosis-prone blocks [19, 38, 51]. TB-silicosis integration might aid in the early identification and treatment of either disease [19]. Early detection of silicosis would allow for earlier implementation of dust control measures in silicosis-prone areas [52–54].

A differentiated TB care strategy identifies a set of critical risk factors implicated in unfavorable treatment outcomes at the time of TB treatment initiation [6, 7, 55, 56]. The current guidelines for differentiated care for people with TB in India recognizes HIV, diabetes, smoking, harmful use of alcohol, and undernourishment as important risk factors for early TB deaths [6]. The present study revealed an association between silicosis and increased mortality and treatment failure rates in people with TB, highlighting the importance of recognizing silicosis as a key comorbidity of TB [6, 8]. Therefore, it is recommended that silicosis should be included as one of the primary risk factors in the differentiated TB care model [6, 8]. Given that chest X-rays are already part of the diagnostic evaluation for TB under the national TB program, early diagnosis and management of silicosis can be made feasible.

This marks the first study to reveal an association between silicosis and TB treatment failure, employing a robust retrospective cohort design, a substantial sample size, and comprehensive analysis, thereby offering crucial insights into TB management in silicosis-prone regions. There are various drawbacks to this study. The current study could not account for several confounding variables, including the existence of a cavity on a chest X-ray, the severity of silicosis, and personal/environmental silica dust exposures. Furthermore, the absence of socioeconomic status (SES) data in this study is a limitation, as SES differences between individuals with and without silicosis could influence treatment outcomes, underscoring the need for future research to address this potential confounder. Another noteworthy limitation is the lack of a more robust baseline nutritional measure, such as body mass index, which should be acknowledged as it may affect the comprehensive evaluation of nutritional aspects and their potential influence on TB outcomes. While the retrospective cohort design establishes temporality through longitudinal tracking, limitations such as potential confounding factors and historical data availability may impact our conclusions. Nonetheless, this study adds to the current body of evidence on the need for active surveillance and management of TB and silicosis in silicosis-prone blocks.

## Conclusions

Controlling silicosis might reduce the mortality rates and treatment failure due to TB in silicosis-prone areas. The coexistence of HIV and silicosis may indicate an increasing trend of TB fatalities in silicosis-prone locations. Higher rates of multi-drug resistant TB in silicosis-prone settings may be contributing to an increase in loss to follow-up. To improve treatment outcomes of TB in silicosis-prone areas, silicosis control activities should be integrated with TB control program. Silicosis should now be acknowledged as a major comorbidity of TB and should be included as one of the key risk factors in the differentiated TB care approach. Primary care physicians should have a high clinical suspicion for silicosis among people diagnosed with TB in silicosis-prone blocks.

## Data Availability

The data for this analysis are available from the author upon a reasonable request and with permission of Gujarat State Tuberculosis Cell (Government of Gujarat, Gandhinagar).

## Abbreviations

aOR: Adjusted Odds Ratio
CI: Confidence Interval
HIV: Human Immunodeficiency Virus
ID: Identification
IQR: Interquartile Range
TB: Tuberculosis
